# Supplementary material for: Risk factors for mortality in COVID-19 patients in sub-Saharan Africa: A systematic review and meta-analysis
Source: PLoS One. 2022 Oct 17;17(10):e0276008. doi: 10.1371/journal.pone.0276008 (PMC9576083; doi:10.1371/journal.pone.0276008)
Supplement: S1 Checklist — (DOCX) [file pone.0276008.s001.docx]

| **Section and Topic** | **Item #** | **Checklist item** | **Reported (Yes/No)** |
| --- | --- | --- | --- |
| **TITLE** | | |  |
| Title | 1 | Identify the report as a systematic review  Risk factors for mortality in COVID-19 patients in sub-Saharan Africa: A systematic review and meta-analysis | yes |
| **BACKGROUND** | | |  |
| Objectives | 2 | Provide an explicit statement of the main objective(s) or question(s) the review addresses.  Mortality rates of coronavirus-2019 (COVID-19) disease continue to increase worldwide and in Africa. In this study, we aimed to summarize the available results on the association between sociodemographic, clinical, biological, and comorbidity factors and the risk of mortality due to COVID-19 in sub-Saharan Africa. | yes |
| **METHODS** | | |  |
| Eligibility criteria | 3 | Specify the inclusion and exclusion criteria for the review.  We included observational studies with Subjects had to be laboratory-confirmed COVID-19 patients; had to report risk factors or predictors of mortality in COVID-19 patients, Studies had to be published in English, include multivariate analysis, and be conducted in the Sub-Saharan region. Exclusion criteria included case reports, review articles, commentaries, errata, protocols, abstracts, reports, letters to the editor, and repeat studies. | yes |
| Information sources | 4 | Specify the information sources (e.g. databases, registers) used to identify studies and the date when each was last searched.  We searched PubMed, Google Scholar, and European PMC between January 1, 2020, and September 23, 2021 | yes |
| Risk of bias | 5 | Specify the methods used to assess risk of bias in the included studies.  The methodological quality of the studies included in this meta-analysis was assessed using the methodological items for nonrandomized studies (MINORS). | yes |
| Synthesis of results | 6 | Specify the methods used to present and synthesise results.  Pooled hazard ratios (HR) or odds ratios (OR) and 95% confidence intervals (CI) were calculated separately to identify mortality risk. In addition, publication bias and subgroup analysis were assessed. | yes |
| **RESULTS** | | |  |
| Included studies | 7 | Give the total number of included studies and participants and summarise relevant characteristics of studies.  Twelve studies with a total of 43598 patients met the inclusion criteria | yes |
| Synthesis of results | 8 | Present results for main outcomes, preferably indicating the number of included studies and participants for each. If meta-analysis was done, report the summary estimate and confidence/credible interval. If comparing groups, indicate the direction of the effect (i.e. which group is favoured).  The results of the analysis showed that the pooled prevalence of mortality in patients hospitalized with COVID-19 was 3%. Older people showed an increased risk of mortality from SARS-Cov-2. The pooled hazard ratio (pHR) and odds ratio (pOR) were 9.01 (95% CI; 6.30-11.71) and 1.04 (95% CI; 1.02-1.06), respectively. A significant association was found between COVID-19 mortality and men (pOR =1.52; 95% CI 1.04-2). In addition, the risk of mortality in patients hospitalized with COVID-19 infection was strongly influenced by chronic kidney disease (CKD), hypertension, severe or critical infection on admission, cough, and dyspnea. | yes |
| **DISCUSSION** | | |  |
| Limitations of evidence | 9 | Provide a brief summary of the limitations of the evidence included in the review (e.g. study risk of bias, inconsistency and imprecision).  The major limitations of the present study are that the data in the meta-analysis came mainly from studies that were published, which may lead to publication bias, and that the causal relationship between risk factors and poor outcome in patients with COVID-19 cannot be confirmed because of the inherent limitations of the observational study. | yes |
| Interpretation | 10 | Provide a general interpretation of the results and important implications.  Advanced age, male sex, CKD, hypertension, severe or critical condition on admission, cough, and dyspnea are clinical risk factors for fatal outcomes associated with coronavirus. These findings could be used for research, control, and prevention of the disease and could help providers take appropriate measures and improve clinical outcomes in these patients. | yes |
| **OTHER** | | |  |
| Funding | 11 | Specify the primary source of funding for the review.  None | No |
| Registration | 12 | Provide the register name and registration number.  None | No |

*From:*  Page MJ, McKenzie JE, Bossuyt PM, Boutron I, Hoffmann TC, Mulrow CD, et al. The PRISMA 2020 statement: an updated guideline for reporting systematic reviews. BMJ 2021;372:n71. doi: 10.1136/bmj.n71

For more information, visit: <http://www.prisma-statement.org/>
