# Supplementary material for: Risk factors for mortality in COVID-19 patients in sub-Saharan Africa: A systematic review and meta-analysis
Source: PLoS One. 2022 Oct 17;17(10):e0276008. doi: 10.1371/journal.pone.0276008 (PMC9576083; doi:10.1371/journal.pone.0276008)
Supplement: S1 Fig — (DOCX) [file pone.0276008.s003.docx]

**Identification of studies via databases and registers**

Records removed *before screening*:

Duplicate records removed (n =5010 )

Records identified from*:

Databases (n = 3426334263)

Registers (n =240 )

**Identification**

Records screened

(n =5010 )

Records excluded**

(n =4890 )

Reports sought for retrieval

(n =120 )

Reports not retrieved

(n =0 )

**Screening**

Reports excluded:108

Reason 1 no outcome of intersst (n =56 )

Reason 2 repeat data(n =20 )

Reason 3 insufficient sample size(n =14 )

Homogeneous population(n=12)

Commentary or editorial(n=6)

Reports assessed for eligibility

(n =120 )

Studies included in review

(n =12 )

**Included**

*Consider, if feasible to do so, reporting the number of records identified from each database or register searched (rather than the total number across all databases/registers).

**If automation tools were used, indicate how many records were excluded by a human and how many were excluded by automation tools.

*From:*  Page MJ, McKenzie JE, Bossuyt PM, Boutron I, Hoffmann TC, Mulrow CD, et al. The PRISMA 2020 statement: an updated guideline for reporting systematic reviews. BMJ 2021;372:n71. doi: 10.1136/bmj.n71

For more information, visit: <http://www.prisma-statement.org/>
