# Supplementary material for: Risk factors for mortality in COVID-19 patients in sub-Saharan Africa: A systematic review and meta-analysis
Source: PLoS One. 2022 Oct 17;17(10):e0276008. doi: 10.1371/journal.pone.0276008 (PMC9576083; doi:10.1371/journal.pone.0276008)
Supplement: Attachment — Submitted filename: RESPONSE TO REVIEWERS.docx [file pone.0276008.s004.docx]

Dear academic editor and reviewers,

Thank you very much for your review. Sincerely all your comments allow us to improve the quality of our manuscript. We did our best to correct every point of comment.

**TO THE ACADEMIC EDITOR**

1. Please ensure that your manuscript meets PLOS ONE's style requirements, including those for file naming. The PLOS ONE style templates can be found at

<https://journals.plos.org/plosone/s/file?id=wjVg/PLOSOne_formatting_sample_main_body.pdf> and

<https://journals.plos.org/plosone/s/file?id=ba62/PLOSOne_formatting_sample_title_authors_affiliations.pdf>.

**We follow the recommendations of the link above for example We Used Level 1 heading for all major sections and Bold type, 18pt font; We cite figures as fig 1, fig 2, etc**

2. Please include a caption for figures 5 to 11.

**We included captions for figures 5 to 11**

3. Please include captions for your Supporting Information files at the end of your manuscript, and update any in-text citations to match accordingly. Please see our Supporting Information guidelines for more information: <http://journals.plos.org/plosone/s/supporting-information>.

**We included captions for supporting Information files at the end of the manuscript and update in-text citations**

4. Thank you for submitting the above manuscript to PLOS ONE. During our internal evaluation of the manuscript, we found significant text overlap between your submission and the following previously published works, some of which you are an author.

- <https://pubmed.ncbi.nlm.nih.gov/34238232/>
- <https://bmcinfectdis.biomedcentral.com/articles/10.1186/s12879-021-06369-0>
- <https://pubmed.ncbi.nlm.nih.gov/33075534/>
- <https://www.frontiersin.org/articles/10.3389/fphys.2021.665064/full>
- <https://link.springer.com/article/10.1007/s11255-020-02740-3?code=aa12583d-3699-4ff6-8f1f-78915123c553&error=cookies_not_supported>
- <https://bmcinfectdis.biomedcentral.com/articles/10.1186/s12879-021-06536-3>

**We changed the majority of sentences in the introduction and results section, we paraphrased sentences in discussions to avoid significant text overlap between our submission and the following previously published works.**

**TO THE REVIEWER**

Results:

1.I believe Table 1 needs to have the time period of study, and perhaps a comment (in table 2 as to the vaccination status during the period of study.

**We add the time period of study and the vaccination status during the period of study in table 2.**

2. Next I am surprised by your mortality rate of 3%. I agree table 1 shows 43598 cases of Covid-19 from the relevant countries but I calculated from your percentage mortality figures and got 2555 deaths which is 5.8%. I think to clarify you need a column that includes the number of deaths as well as the mortality percent.

**Thank you, dear reviewer, for making this calculation. Indeed, our mortality of 3% was wrong. And after reviewing in depth the mortality rates of all countries, we also found that there was a transcription error of the mortality rate values in the Jaspard et al study which was actually 5% instead of 29% (see revised table 1). And we added a column for the number of deaths as recommended and after recalculating the total number of deaths was 2103, recalculating the mortality rate is 4.8%. That's why I didn't calculate with the software anymore and removed the figure number 2 but calculated as you did to be more sure. Thank you very much for this**

3. Table 1 should probably include also the source of the cohort of cases - eg community, hospital admitted and I think the mortality is always going to be different.

**We have included in Table 1 the source of the cohort of cases. And indeed, the mortality was different depending on whether the case was hospitalized or community**

4. Table 2 does try to describe the source of cases included. I think you need to separate the mortality of cases treated in the community from those treated in hospital. This would explain some of the significant variation in your mortality rates.

**Table 2 describes the source of cases included in the column of setting**

5. Risk factors: I thought this was a very informative section.

**Thank you**

Discussion:

6. From the countries included in the twelve studies what are the WHO reported numbers and mortality rates from these cases and how do they differ from the results in your meta-analysis and why?

**We compared the mortality obtained in our included studies and the mortality of the countries from which these studies came according to WHO, and we found huge differences in countries where the cases were only hospitalized but not a big difference in countries where community cases were also included.**

**Below you can see the paragraph we added in the discussion:**

**The mortality prevalence of 4.8% in our study is higher than the mortality reported by the WHO. The reason for this is that the majority of the included patients in our meta-analysis were hospitalized, and in some included studies, the proportion of severe and critical patients was very high. The emergence of variants of concern may also influence case numbers and mortality, although almost all of the included studies were conducted in the first wave when these variants were not yet formally reported. Patient mortality in the DRC ranged from 13-29% versus 1.5% according to WHO data. In Ghana, mortality was 16% versus 0.87% according to WHO data. In Nigeria, mortality ranged from 3.3 to 9% versus 1.2% according to WHO data. In these countries, our meta-analysis shows high prevalence because most of the included patients are hospitalized, with a high proportion of severe cases. Mortality in South Africa and Ethiopia was close to WHO data (0.8 and 2.8 versus 2.5 and 1.5) because the studies included in these two countries also included cases from the community [1]**

Dr Ben Bepouka

Corresponding author
