# Supplementary material for: Risk factors for mortality in COVID-19 patients in sub-Saharan Africa: A systematic review and meta-analysis
Source: PLoS One. 2022 Oct 17;17(10):e0276008. doi: 10.1371/journal.pone.0276008 (PMC9576083; doi:10.1371/journal.pone.0276008)
Supplement: Attachment — Submitted filename: Response to Reviewers.docx [file pone.0276008.s005.docx]

Dear academic editor and reviewer, thank you very much for your comments and corrections which help to improve the quality of our manuscript.

To the reviewer:

Find the different corrections we could make according to your remarks:

Study selection P14: There is an error in your numbers for the cases found and excluded: "found 34263 articles. After eligibility criteria, 34491 were excluded" - you have excluded more articles than you found. There is a miscalculation here.

**it is true that there was an error in the calculation, the corrected sentence is:**

**In the PRISMA flowchart, we can see the approach used for the document search. Initially, we found 34263 articles. After eligibility criteria, 34251 were excluded. Of the excluded studies, 5010 articles were recorded after duplicates were removed, 4890 were perceived as unrelated searches, and 108 articles met the exclusion criteria. Finally, 12 published articles were included (Fig 1).**

Table 2: Given none of the studies reported vaccination status, I suggest you change the table title to risk factors and leave out the vaccination status from the actual table (originally, I thought you may have a mix of vac status but all studies did not provide this). I don't think the vaccination status column is relevant given you have stated in the text and could do in a table footnote that none of the studies reported vaccination status. This can then be discussed as it would become increasingly relevant now that vaccinations are available.

**We removed the vaccination status from the title and the table and we have placed a table footnote that none of the studies reported vaccination status. And we discussed this in the discussion like this:**

**None of the studies reported vaccination status. The included studies were conducted in the first wave when vaccination was not yet effective. Vaccination could help reduce this high inpatient mortality, especially if vaccination coverage was high. In the literature, there is evidence of the efficacy of COVID-19 vaccines against severe forms and a reduction in mortality. Although most doses of COVID-19 vaccines have been administered in high and middle-income countries. The efficacy of the vaccines has been demonstrated in several countries where, for example, the efficacy of the two doses was 92%, 96%, and 89%, respectively, in Israel, the USA, and the UK** **[50]. But in Africa, up to the end of 2021, vaccination coverage was only 17% [51].**

I realise that some of the papers included both hospitalised and community patients and perhaps did not state what the mortality of each group was separately.
I still think it would be worth in the text stating what the mean mortality and range was of the patients who were hospitalised and, if possible, those that were known only to be treated or diagnosed in community but did not require admission. You will need to leave some studies out of this calculation but your hospitalised patient only studies suggest the mortality ranges from 4-29% which means hospitalisation = much higher mortality rate than 4.8%. This would be worth commenting on.

**Dear Reviewer, you are correct; the articles that included both inpatients and community patients did not report the mortality of each group separately.**

**In the text we stated:**

**The range of inpatient mortality varied from 4% to 29%, with the mean mortality of the patients who were hospitalized of 7.3% (439/5972). The range of mortality for the combined studies (combining inpatients and community-diagnosed patients) varied from 0.8 to 9%, with a mean mortality rate of 4.4% (1664/37626). Separate data on the mean and range of mortality of patients treated or diagnosed in the community but not requiring admission were not clearly available in the included studies.**

**In the discussion, we added the sentence:**

**In addition, considering overall mortality was 4.8%; The range of inpatient mortality was 4–29% with a mean inpatient mortality of 7.3% (439/5972). Considering the range of mortality being 4–29%, one would expect higher mortality than 4.8%. This value of 4.8% is due to the fact that it is overall mortality combining inpatients and community patients, this value of 4.8% is due to the fact that it is the overall mortality combining inpatients and community patients, whose number is not negligible.**

Table 1: I make the mortality rate from your hospitalised only studies 7.3% (439/5972) but some of the combined studies may also have stated the mortality rates for hospitalised and community patients separately.
I would suggest reorganising table 1 to show all the hospitalised only studies separately with totals compared with the combined studies.

**We reorganized Table 1 by presenting all inpatient-only studies separately, comparing the totals with the combined studies.**

To the academic editor:

the references added to the discussion section are the following:

50. Tregoning JS, Flight KE, Higham SL, Wang Z, Pierce BF. Progress of the COVID-19 vaccine effort: viruses, vaccines and variants versus efficacy, effectiveness and escape. Nat Rev Immunol. 2021 Oct;21(10):626-636. doi: 10.1038/s41577-021-00592-1. Epub 2021 Aug 9. PMID: 34373623; PMCID: PMC8351583.

51. WHO Africa faces 470 million COVID-19 vaccine shortfall in 2021. 2021. [https://www.afro.who.int/news/africa-faces-470-million-covid-19-vaccine-shortfall-2021. Accessed August 28](https://www.afro.who.int/news/africa-faces-470-million-covid-19-vaccine-shortfall-2021.%20Accessed%20August%2028), 2022.
